# Lumen-apposing metal stent gastrojejunostomy for biliary drainage of an obstructive biliodigestive anastomosis: an endoscopic resolution avoiding surgery

**DOI:** 10.1055/a-2789-2172

**Published:** 2026-03-16

**Authors:** Ottavia De Simoni, Irma Albergati, Carola Cenzi, Pierluigi Pilati, Mario Gruppo, Alberto Fantin

**Affiliations:** 1 90383 Unit of Surgical Oncology of the Digestive Tract, Veneto Institute of Oncology IOV-IRCCS, Padua, Italy; 2 90383 Unit of Gastroenterology, Veneto Institute of Oncology IOV-IRCCS, Padua, Italy; 3 90383 Clinical Research Unit, Veneto Institute of Oncology IOV-IRCCS, Padua, Italy

A 76-year-old man with a long-standing history of recurrent cholangitis was referred to our department. Twenty years earlier, he had undergone surgery to remove a large hepatic cyst in segments S5–S6. The postoperative course was complicated by multiple reoperations for choleperitoneum, ultimately requiring the creation of a biductal hepatico-jejunostomy anastomosis.

**Fig. 1:**
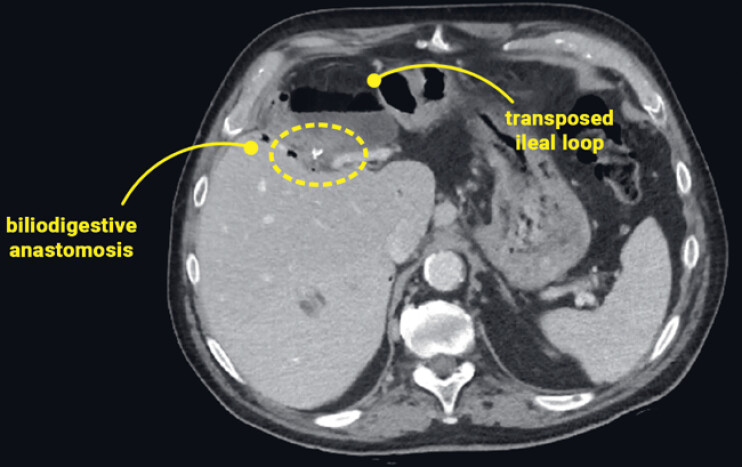
A preoperative abdominal CT scan showing the dilation of the afferent tract of the transposed loop with suspected impaired biliodigestive anastomosis discharge. CT, computed tomography.

An abdominal computed tomography (CT) scan revealed the dilation of the afferent tract of the transposed jejunal loop, suggesting impaired drainage through the biliodigestive anastomosis ( Fig. 1 ). Although surgical revision was deemed technically feasible, it was associated with considerable operative complexity and high perioperative risk. Haase et al. described a case of an obstructed biliodigestive anastomosis in a patient with a progressive pancreatic cancer, in which the transgastric endoscopic ultrasound-guided placement of a lumen-apposing metal stent (LAMS) into the afferent jejunal was performed with good results 1 .

**Fig. 2:**
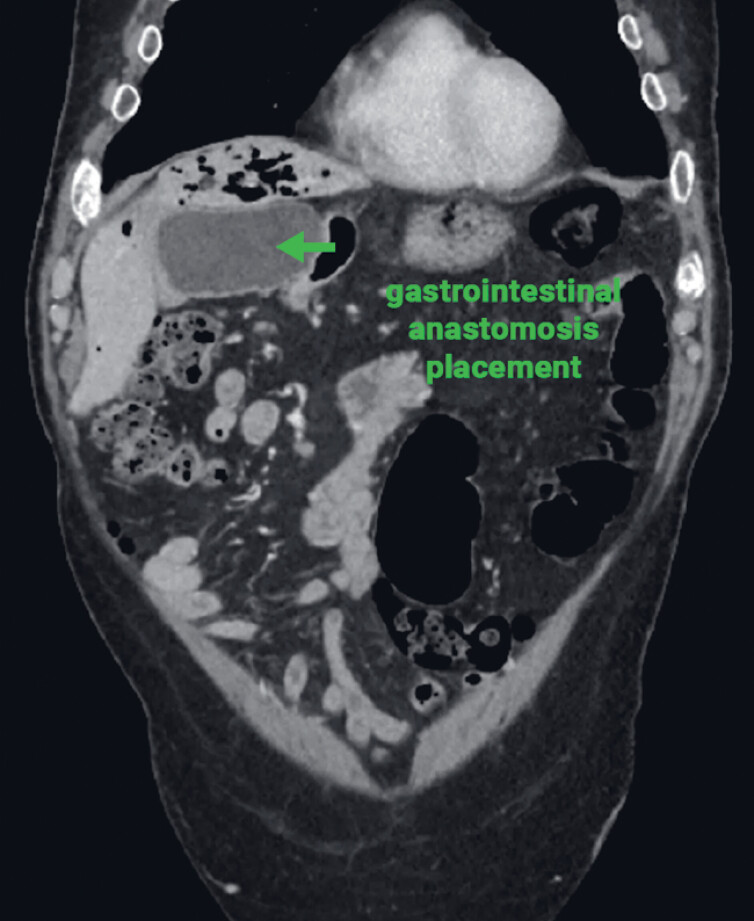
A preoperative CT scan showing the feasibility of the endoscopic ultrasound-guided placement of a LAMS between the gastric antrum and the afferent jejunal loop. CT, computed tomography; LAMS, lumen-apposing metal stent.

**Fig. 3:**
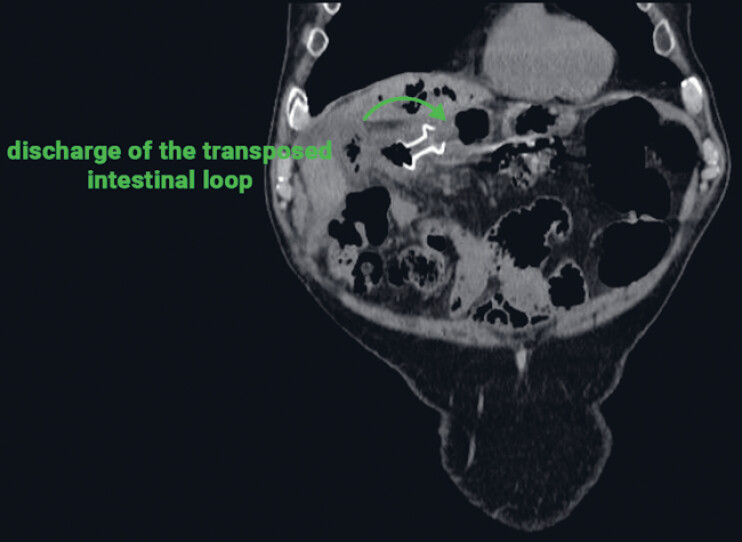
The final position of the stent between the prepyloric region and the afferent loop, demonstrating effective bile drainage into the stomach.

For this reason, an endoscopic approach was considered ( Fig. 2 ). The patient subsequently underwent the transgastric endoscopic ultrasound-guided placement of a lumen-apposing metal stent (LAMS) into the afferent jejunal loop ( Fig. 3 ), as shown in Video 1 .

Lumen-apposing metal stent gastrojejunostomy is performed for biliary drainage of an obstructive biductal hepatico-jejunostomy anastomosis.Video 1

A CT scan performed 1 month after the procedure confirmed correct stent positioning, adequate biliary drainage through the anastomosis, and a marked reduction in intrahepatic bile duct dilation.

Approximately 1 year later, he reported two brief, self-limited episodes of fever. Endoscopic ultrasound showed the partial obstruction of the LAMS at the prepyloric site due to food debris. The material was removed, restoring unobstructed bile flow through the stent. Follow-up remained uneventful, and the patient remained asymptomatic.

This case highlights the feasibility, safety, and efficacy of LAMS placement as a minimally invasive alternative for managing impaired bile drainage following complex biliary surgery. In this patient, the endoscopic approach successfully resolved recurrent cholangitis, improved the quality of life, and, most importantly, avoided a highly complex surgical procedure with a considerable perioperative risk.

Endoscopy_UCTN_Code_TTT_1AS_2AD
